# Longitudinal associations of age and prenatal lead exposure on cortisol secretion of 12–24 month-old infants from Mexico City

**DOI:** 10.1186/s12940-016-0124-1

**Published:** 2016-02-29

**Authors:** Marcela Tamayo y Ortiz, Martha María Téllez-Rojo, Rosalind J. Wright, Brent A. Coull, Robert O. Wright

**Affiliations:** National Council of Science and Technology, Center for Nutrition Research and Health, National Institute of Public Health, 7a. Cerrada de Fray Pedro de Gante # 50, Col. Sección XVI Tlalpan, C.P. 14080 México D.F., Mexico; Center for Research in Nutrition and Health, National Institute of Public Health, Universidad No. 655, Col. Santa María Ahuacatitlán, Cerrada los Pinos y Caminera, CP. 62100 Cuernavaca, Morelos Mexico; Departments of Pediatrics and Preventive Medicine, Pediatrics Kravis Children’s Hospital, New York, USA; The Mindich Child Health & Development Institute, Icahn School of Medicine at Mount Sinai, Annenberg Building Floor 14th Floor Room 8, 1468 Madison Avenue, New York City, NY 10029 USA; Department of Biostatistics, Harvard T.H. Chan School of Public Health, 655 Huntington Avenue, Building II, Room 413, Boston, MA 02115 USA; Department of Preventive Medicine, Icahn School of Medicine at Mount Sinai, 17 East 102 Street Floor 3 West Room D3-110, New York City, NY 10029 USA

**Keywords:** Lead, Cortisol, Epidemiology, Prenatal

## Abstract

**Background:**

Cortisol has functions on homeostasis, growth, neurodevelopment, immune function and the stress response. Secretion follows a diurnal rhythm that mediates these processes. Our objective was to examine the association between prenatal lead exposure and infant diurnal cortisol rhythms.

**Methods:**

We measured infant cortisol rhythms in saliva collected repeatedly over 2 days at either 12 (*n* = 255) or 18–24 (*n* = 150) months of age. Prenatal lead exposure was assessed by measuring maternal pregnancy blood lead levels and early postnatal maternal bone lead content. We analyzed age-specific basal secretion and the association between trimester-specific and cumulative lead exposure with a) change in total diurnal cortisol and b) the shape of the cortisol curve across the length of the day.

**Results:**

Our results showed age related differences in salivary cortisol secretion and an age dependent association with maternal lead exposure. In age-stratified models we saw an inverse association between lead and cortisol levels in 12-month-old infants and a positive association for 18–24-month-old infants. For the 12-month-old infants 2nd-trimester-lead ≥10 μg/dL was associated with 40 % lower cortisol levels (95 % CI (−57, −16)) and a significant change in the shape of the cortisol curve (*p* = 0.01), compared to infants with low blood lead levels (<5 μg/dL).

**Conclusions:**

Basal cortisol secretion changes with age. Increased early gestation lead exposure alters diurnal cortisol rhythms and the association is modified by infant age, perhaps representing an early maturation of cortisol homeostasis.

**Electronic supplementary material:**

The online version of this article (doi:10.1186/s12940-016-0124-1) contains supplementary material, which is available to authorized users.

## Background

Endocrine disruption refers to environmental factors that interfere with hormone metabolism and homeostasis, processes that might explain developmental programming by prenatal chemical exposure [1, 2] ultimately impacting adult health and disease [3]. Cortisol is a critical metabolic hormone that mediates several homeostatic processes that are essential to development and plays a direct role in neurodevelopment, growth/obesity and immune function. The hypothalamic-pituitary-adrenal (HPA) axis is the primary regulator of cortisol metabolism and its basal secretion; it mediates both stable day-to-day physiologic secretion of cortisol, and the increase in secretion that arises from environmental stimuli (danger, infection, diet, etc.) [4]. Understanding how the environment might program alterations in cortisol physiology requires measuring cortisol noninvasively (to avoid the stress of blood draws that can confound results), and repeatedly (to capture the diurnal variation in cortisol levels that naturally occurs).

Prenatal lead (Pb) exposure has been consistently associated with a number of adverse health effects that overlap with cortisol function, including growth [5, 6] and altered neurodevelopment [7–9]. Research on environmental chemical toxicants and their effects on infant or early childhood HPA axis functioning which regulates cortisol excretion [10–12] remains limited. Studies on lead exposure and cortisol have mixed results, showing an increase or no change in cortisol production in 9 year old children and in occupationally exposed to lead male workers respectively [13–15]. Nevertheless, studies examining associations with exposure to other environmental chemicals in early development have suggested an increase in cortisol production and also suggest that effects may vary based on developmental stage of exposure and effect measurement of HPA function [16]. The present study expands upon this prior research by examining the relationship between prenatal exposure to Pb and *basal* HPA axis functioning (rather than the response to a stressor), as characterized by infant diurnal cortisol rhythms measured using timed salivary samples, in 12–24 month-old infants in a Mexican birth cohort. We are unaware of any data that establish norms for basal cortisol rhythms for these ages, and there is relatively little data on the timing of HPA axis maturation (although there is evidence of the establishment of the basic cortisol pattern (higher levels in the morning and lower levels late in the day) by 1 year of age, the current hypothesis is that it occurs around the 3rd year of life in humans [17]. Therefore, our main objectives were to investigate: *a)* if there is an age-dependent difference in the association of lead with cortisol rhythms, *b)* the association between lead and infant diurnal cortisol rhythms, and we hypothesized that higher prenatal lead exposure would be associated with increased cortisol production over the course of the day and *c)* if a specific window of prenatal susceptibility to lead could be identified. For objectives *a* and *c*, we hypothesized that there would be a difference in the association depending on infant age and that a specific prenatal window of exposure would be detected, however we preferred not to define a direction or magnitude of the effect (objective a) or to point at a specific window (objective c) due to lack of human studies on this specific theme.

## Methods

### Study population

This study was conducted in the Programming Research in Obesity, Growth, Environment and Social Stressors (PROGRESS) birth cohort in Mexico City, previously described in more detail [18]. Briefly, women were invited to participate during their prenatal care visits at 4 clinics belonging to the Mexican Social Security System (Instituto Mexicano del Seguro Social [IMSS]) and were consented in a face-to-face interview and enrolled in their 2nd trimester of pregnancy. Between 2007 and 2011, 948 live infants were born and 760 (80 %) mother-infant pairs returned for follow-up visits between 6 and 24 months of age. The saliva collection kit was provided to all participants in follow-up (between 12 and 24 months) and the protocol was completed by 411 children of which 405 were included in the analyses. Participant characteristics did not differ significantly to those of non-participants (*p* > 0.05 for all comparison tests of covariates between non-participants and participants) as shown in Table 1. Study protocols were approved by the institutional review boards of the Icahn School of Medicine at Mount Sinai, Harvard T. H. Chan School of Public Health, the National Institute of Public Health Mexico, the Mexican Social Security System, and the National Institute of Perinatology, Mexico. At each visit the study protocol was explained to women, who provided informed consent before any procedure was carried out.

**Table 1:** Comparison of Non-Participants and Participants

	Non-participants	12 month-old infants	18–24 month-old infants
	*n = 355*	*n = 255*	*n = 150*
Mother			
Age at delivery (years)	26.7 ± 5.5	27.1 ± 5.6	27.5 ± 5.1
Education (total years)	11.8 ± 2.8	11.9 ± 2.8	11.6 ± 2.8
Pre-pregnancy BMI (kg/m^2^)	24.9 ± 4.1	25.8 ± 4.4^*^	24.7 ± 3.9
2nd trimester blood lead (μg/dL)^a^	3.6 ± 2.5	3.5 ± 2.5	3.9 ± 2.8
3rd trimester blood lead (μg/dL)^b^	3.8 ± 2.7	3.7 ± 2.9	4.2 ± 3.4
Tibia lead (μg/g)^c^	4.7 ± 5.4	5.6 ± 5.8	4.9 ± 5.0
Infant			
Sex (male) *n* (%)	182 (51)	129 (51)	90 (60)
Ever breastfed *n* (%)^d^	303 (85)	224 (88)	139 (93)
Gestational Age, weeks	38.3 ± 1.9	38.3 ± 1.5	38.5 ± 1.6
Birth Weight, kg	3.0 ± 0.5	3.1 ± 0.4	3.2 ± 0.4^*^

For all analyses the imputed values for missing blood and tibia lead were used

^a^non-participant *n* = 354, 18–24 month-old infants *n* = 149

^b^non-participant *n* = 301, 12 month-old infants *n* = 229, 18–24 month-old infants *n* = 139

^c^non-participant *n* = 270, 12 month-old infants *n* = 199, 18–24 month-old infants *n* = 117

^d^non-participant *n* = 329, 12 month-old infants *n*=, 18–24 month-old infants *n* = 145

*Unless noted *p* > 0.05 for all comparison tests of covariates between non-participants and each age group

### Maternal lead in blood

Venous blood from women was drawn into royal blue trace metal vacutainer (Becton-Dickinson and Company, Franklin Lakes, New Jersey) tubes containing EDTA. Samples were collected from women in the second trimester (2 T) between 16 and 20 weeks of gestation and the third trimester (3 T) between 30 and 34 weeks. Samples were kept at 4 °C until analyzed. BPb concentrations were measured using a dynamic reaction cell inductively-coupled plasma mass spectrometer (Elan 6100; PerkinElmer, Norwalk, CT). Five replicate measurements of each sample were taken and averaged. The recovery of the analysis quality control standards and spike samples was 90–110 %, and the limit of detection for the procedure was 0.02 μg/dL.

### Maternal bone lead

To assess cumulative Pb exposure in mothers we used tibia bone Pb levels (half-life of approximately 10 years [19]) during the 1 month postpartum visit. Previous research demonstrates that pre-pregnancy bone lead levels are highly correlated with 1 month postpartum levels [20, 21]. Maternal bone Pb was measured in the mid-tibial shaft using a K-shell X-ray fluorescence instrument (KXRF) [22]. Each leg was measured for 30 min, and the results from both legs were averaged, weighted by the inverse of the measurement variance. Negative bone Pb values were replaced by sampling from a uniform distribution over the range of 0 to the DL (2 μg/g), as previously described [21, 23, 24].

### Infant saliva collection

The saliva collection protocol (illustrated with photographs) was explained to mothers during the 12 month study visit. The collection material, an information diary and a printed copy of the protocol was provided to all participants who attended their study visit. If a mother did not participate in the saliva collection following this visit, we inquired about participation at both 18 and 24 months as well. A total of 411 children provided saliva for cortisol at only one of these three visit dates (54 % completion rate).

Mothers were asked to collect 4 saliva samples per day from their child at home (*early morning:* after the infant woke up and had a diaper change but before breakfast, *mid-morning:* between 11:00 am and 1:00 pm, *mid-afternoon:* between 3:00 and 5:00 pm and *night:* at least 30 min after dinner, before bed time) for 2 days (8 samples total), and instructed that: collection days did not have to be consecutive but no more than a week apart, to wait at least 30 min before collecting the sample if the infant had been fed (including breastmilk), to collect samples on “typical” days (not days that were foreseen to be particularly stressful or busy), not to collect the samples if the infant was ill, taking medicine or had an allergic reaction to any food or insect bite.

Saliva was collected using a cotton braid which the infant was allowed to bite and suck between 10 and 30 s. Saliva was then extracted using a needleless syringe to previously labeled collecting tubes on which the exact collection time was registered by the mother.

In the diary provided mothers were asked to record the specifics of collection for each sample, namely: the date for each collection day and times for the collection of each sample as well as for infant wake up, breakfast, dinner and bedtime. In all 8 times were recorded regarding the samples. Information on the child’s sleep (whether the child slept enough (as usual) and if any naps were taken during the day registering the time of day and duration), and health (illness, medication name and time administered) particular to the sample collection day were also recoded.

Samples were stored in the participant's refrigerator until they were collected by our staff and then frozen at −70 °C until shipment to the laboratory of the Technical University of Dresden, Germany for cortisol analysis. Saliva samples were analyzed in duplicate using a chemi-luminescence-assay with a sensitivity of ~0.16 ng/ml (IBL; Hamburg, Germany, Clemens Kirschbaum). Control samples covering at least three levels of cortisol were run for each day. The intra- and inter-assay coefficients of variation were less than 8 %.

We examined all the participants cortisol curves carefully, looking for any aberrant pattern. If we detected any irregularities, we consulted the diary for information on feeding, sleep or illness and excluded the sample(s) from our analyses if considered it relevant. Samples were also excluded if cortisol levels were greater than 3 SD above or below the mean, when fever was reported and those for a 35 months-old infant. Only sampling days with at least 3 samples and with the wake up time registered were considered for the analyses. Our final sample included 3,110 cortisol samples (96 % of total collected samples) from 405 infants.

### Covariates

Demographic information was obtained through standardized questionnaires. Potential covariates were identified based on the existing literature on Pb and cortisol response [25] as well as prior studies of cortisol rhythms performed in non-environmental health aims [17, 26, 27]: child’s sex, gestational age, birth weight and breast-feeding (ever/never), maternal age at delivery, education (total years of school), pre-pregnancy BMI (self-reported weight and use of 2 T height), and weekday vs. weekend sample collection. Covariates were left out of the analyses if they were not associated with the exposure or outcome (*p* > 0.1) in bivariate models or if the cortisol effect estimates were not changed by more than 10 %. All models were adjusted for child’s sex and maternal age at delivery, education and pre-pregnancy BMI.

### Statistical analysis

We used longitudinal functional mixed effects regression models with penalized splines as described by Sanchez and colleagues [28]. This modeling framework accounts for the non-linearity of cortisol rhythms over the course of a day and addresses the multilevel structure in the data, whereby repeated measurements are taken within a day and measurements from multiple days are recorded for each participant [28]. The resulting models reflect how the association between exposure and cortisol levels varies as a function of “time since awakening” linked to a given cortisol measurement. Models can be run that assume that this association is constant across the course of the day “*constant effect model*” (CE), which implies that exposure is associated with a shift (increase or decrease) in the overall mean cortisol curve while maintaining the curve’s shape. Alternatively, the model can assume that the association between exposure and cortisol level depends on the time since awakening “*time-varying-effect model*” (TVE), which allows changes in the shape of the cortisol curve. For the TVE model, results are illustrated graphically to present how the curve changes shape as a function of exposure. More details can be found in the Additional file 1. Cortisol concentrations were skewed to the left therefore we log-transformed them to better satisfy the normality assumption of the models.

**Fig. 1:**
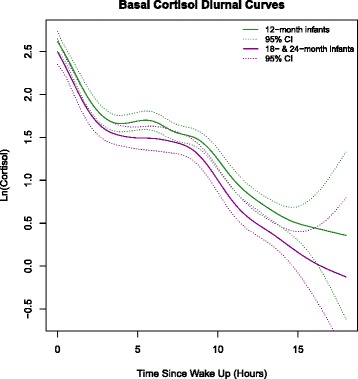
“Unadjusted Basal Cortisol Rhythms for 12- and 18–24 month-old infants”. Mixed effects regression model with penalized splines using age group as a main predictor

In line with our aims, our initial analysis considered age as primary predictor of cortisol rhythms and this was statistically significant (*p* > 0.001). We then plotted the unadjusted cortisol curves for 12, 18 and 24 month old infants separately. Those of the 18- and 24-month old infants were very similar in shape and concentrations and not statistically different. Average cortisol levels in the 12-month old group were 21 % higher (95 % CI (9, 34)) than those of the 18–24-month infants (Fig. 1). Next, we ran models with an interaction term Pb x exact-age-at-collection (we calculated the child’s age in days using the difference between the birth date and the date when the saliva was sampled) which was statistically significant. Therefore, we subsequently ran separate prediction models for infants at 12 months and grouped 18–24 month old infants.

We defined cut-points to assess nonlinear relationships and whether an exposure threshold for Pb may be driving the associations. For bone Pb, we used tertiles and for BPb, we carried out analyses based on the updated CDC reference level for BPb of 5 μg/dL and the former level of 10 μg/dL, which is the current reference level in Mexico according to the health norm NOM-199-SSA1-2000. We defined 3 categories of BPb exposure (“*Lower*” (Reference): <5 μg/dL; “*Moderate*”: 5 ≤ Pb <10 μg/dL; and “*Higher*”: Pb ≥10 μg/dL).

In order to determine a prenatal window of susceptibility for Pb we regressed cortisol on 2 different BPb indicators (2nd trimester -2 T BPb and 3rd trimester-3 T BPb) and an indicator of cumulative Pb exposure (maternal tibia bone Pb levels).

To account for missing Pb indicator data (see Table 1 for number of samples for each indicator), we used multiple imputation. We generated 10 data sets, which we used to run each of the models. For each model, the results of 10 imputations were averaged to give the final effect estimates. Standard errors were calculated using the methods that combine the within and between-imputation uncertainty. We ran all our models with and without imputation and the results for the imputed models were consistent with the direction and magnitude of the associations. For the statistical analyses we used SAS 9.3 (SAS Institute, Inc., Cary, North Carolina, proc MI with the MCMC method for multiple imputation) and R version 3.0.2 with the gamm () function from the mgcv package for the functional mixed models [28].

## Results

Age-dependent cortisol levels were confirmed by the results from the mixed effects regression model with penalized splines using age group (12- and 18–24-month-olds) as a main predictor. As reported above, younger children had higher basal cortisol levels throughout the day (Fig. 1). We found no statistically significant difference in saliva collection times between age groups. More information on cortisol concentrations by sample and for saliva collection times can be found in Table 1 of the Additional file 1.

**Fig. 2:**
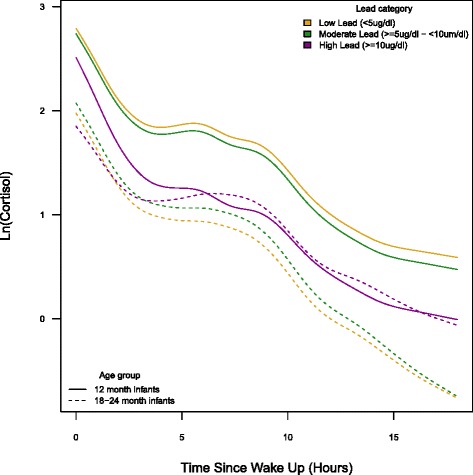
“Effect of 2nd pregnancy trimester blood lead in categories on diurnal ln(cortisol) curves stratified by age group (12 and 18–24-month-old)”. All models were adjusted for child’s gender and maternal age at delivery, pre-pregnancy BMI and total school years

Results from *Constant Effect Models* (lead exposure not influencing the shape of the cortisol curve throughout the day) showed that among the 12-month old infants, mean cortisol levels for the moderate (5 ≤ Pb <10 μg/dL) and higher (Pb ≥10 μg/dL) Pb exposure groups were lower compared to those in the referent category of <5 μg/dL across BPb indicators (Table 2). The mean cortisol level for the higher-2 T-BPb was estimated to be 40 % lower (95 % CI −57 %, −16 %) than the mean cortisol level for the 2 T-BPb referent group. Moderate-3 T-BPb was marginally associated with a 13 % lower mean cortisol level (95 % CI −27 %, 3 %) compared to the mean cortisol level for the 3 T-BPb referent group. This was contrary to our hypothesis that lead would be associated high higher cortisol levels. However, among the 18–24 month olds the associations between Pb exposure and cortisol were generally positive, although not statistically significant. Our results are clearer when presented graphically. As an example, Fig. 2 shows the results for the categorical 2 T-BPb models stratified by age group. The adjusted natural log cortisol (lncortisol) curves illustrate the direction and magnitude of the associations with respect to the mean cortisol curve for each exposure category and for each age group.

**Table 2:** Categorical analyses of the change on total diurnal infant (ln) cortisol level stratified by infant age group

	Lower Lead *(<5 μg/dL)*	Moderate Lead *(5 ≤ Pb < 10 μg/dL)*		Higher Lead *(≥10 μg/dL)*	
		*β*	*% change*	*β*	*% change*
*12-Month Infants* ^*a*^					
2nd trimester	Ref	−0.07 (−0.24, 0.10)	−7 (−22, 10)	−0.51 (−0.85, −0.18)*	−40 (−57, −16)*
3rd trimester	Ref	−0.14 (−0.31, 0.03)	−13 (−27, 3)	−0.02 (−0.31, 0.26)	−2 (−26, 30)
Tibia^c^	Ref	0.02 (−0.14, 0.19)	2 (−13, 20)	−0.03 (−0.21, 0.14)	−3 (−19, 15)
*18- & 24-Month Infants* ^*b*^					
Second Trimester	Ref	0.11 (−0.08, 0.30)	12 (−8, 35)	0.23 (−0.19, 0.65)	26 (−18, 92)
Third Trimester	Ref	0.01 (−0.17, 0.20)	1 (−16, 22)	−0.05 (−0.51, 0.41)	−5 (−40, 51)
Tibia	Ref	0.10 (−0.13, 0.32)	10 (−12, 38)	0.14 (−0.08, 0.35)	14 (−8, 42)

Effect estimates: β *(95 % CI)*, % change and *(95 % CI)*. All models are adjusted for child’s gender and maternal age at delivery, pre-pregnancy BMI and total school years

^a^ *n* = 255

^b^ *n* = 150

^c^tertiles were used for tibia bone (μg/g)

*p < 0.05, remained significant (*p* < 0.025) after Bonferroni correction for multiple comparisons

**Fig. 3:**
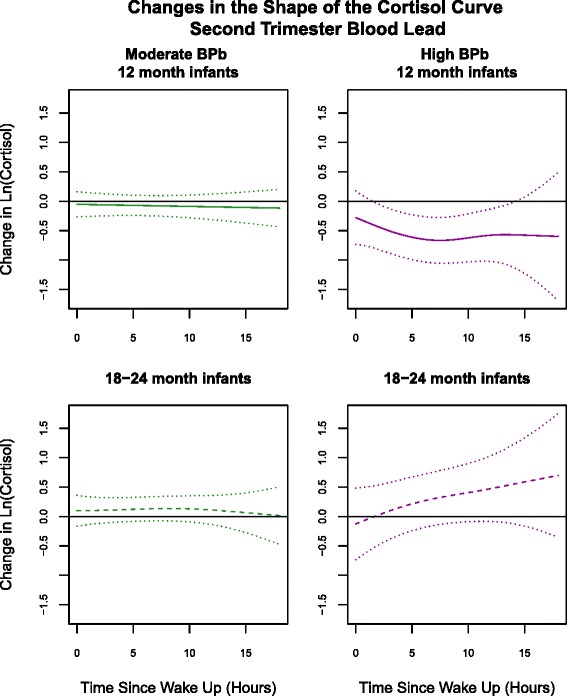
“Change in ln(cortisol) throughout the day associated with moderate *(5 ≤ Pb < 10 μg/dL)* and high *(≥10 μg/dL)* 2nd trimester blood lead by age group”. All models were adjusted for child’s gender and maternal age at delivery, pre-pregnancy BMI and total school years

Figure 3 shows the results for the Time Varying Effect (TVE)-models stratified by age, using the indicator for 2 T-BPb. These models allow for lead exposure to influence the cortisol level at any time point throughout the day, resulting in a change in the shape of the curve. The curves in Fig. 3 illustrate the estimated differences in mean (lncortisol) curves for the moderate-Pb and high-Pb exposure groups, each relative to the low-Pb exposure group. For infants in both age groups, the mean curves associated with moderate-2 T-BPb exposure are the same *shape* as that for the corresponding low exposure group (no departure from the 0-change line). However, for both age groups the *shape* of the mean curve associated with high-2 T-BPb exposure is modified relative to that for the low exposure group, with smaller differences occurring at the beginning of the day and larger differences occurring later in the day. This difference is significant (*p* < 0.05) among the 12-month-olds around midday (5–10 h post awakening). As in CE-models, these associations are inverse for the 12-month-olds and positive for the 18–24-month-olds. The results from models based on the other maternal Pb indicators were similar (data not shown).

Lastly, we confirmed that there might be a relevant *window of susceptibility* to lead and HPA axis development. The stronger and statistically significant association seen with 2 T BPb, specifically with higher BPb levels point at the susceptibility of this gestational stage for subsequent cortisol metabolism in infants.

## Discussion

In this prospective birth-cohort study we found higher basal cortisol levels in 12 month-olds relative to 18–24 month-olds which most likely represents the normal development of the HPA axis [17]. This information alone will be critical in planning future research on cortisol homeostasis as investigators should consider age at collection. Our results show that Pb exposure in *pregnancy* is associated with the infant cortisol curve differently based on the age at which the rhythm is measured. BPb was associated with a *downshift* in the cortisol curve at 12 months but with an upward shift in the cortisol curve at 18–24 months. Keeping in mind that the normal age trend from 12- to 18–24 months is a downshift, Pb exposure appears to produce a cortisol rhythm pattern in 12 month olds that is similar to the pattern seen in 18–24 month olds. This would be biologically-consistent with Pb driving a premature maturation of cortisol rhythm. Also, it appears that among 12-month-olds, higher prenatal BPb exposure modifies the shape of the cortisol rhythm. Among lower exposed children an age-dependent shift in the cortisol profile is seen and is likely a normal transition, but this shift is different if *prenatal* BPb is above 10 μg/dL.

Cortisol is critical to neurogenesis and given Pb’s well-known effects on brain development, these results would be consistent with cortisol disruption being a mechanism mediating Pb’s neurotoxicity, a hypothesis that has been studied in animal models. Corticosterone levels in rodent pups were altered by prenatal exposure to Pb in early pregnancy [29–32], but not when Pb exposure occurred in later gestational stages [33]; Pb accelerated age-related reductions in pre-stressor corticosterone levels in adult animals [29]; and there was a dose-response relationship of prenatal Pb with cortisol levels [29]. Two other human studies have examined the adrenocortical *response* to stress in the context of Pb exposure, rather than basal cortisol rhythms and generally included older participants with presumably mature HPA axis function [13, 15]. In Gump et al. [14], investigators examined the association between prenatal and early childhood exposure to Pb and the cortisol response to an acute physical stressor in 9 year-old children. Both cord blood Pb and 2-year-old BPb measures were associated with an increased cortisol response to a cold immersion stress. Fortin et al. [13] examined the response to a psychological stressor among occupationally Pb exposed adult men. They found that higher Pb exposures were associated with lower basal cortisol levels [13]. Our results complement these findings, despite their methodologic differences, when taken as a whole, all indicate an association between Pb exposure and HPA axis disruption.

Our findings are in line with previous studies suggesting that Pb toxicity includes endocrine disruption. Given the role of cortisol in so many physiologic systems, the pleiotropic effect of Pb on other developmental processes, such as growth, cardiovascular and immunologic function, may also be related to disruptions of HPA axis function or maturation. Of note, the hippocampus, a well-known target of Pb toxicity and a central anatomic mediator of memory formation, has the highest concentration of glucocorticoid receptors in the central nervous system.

### Limitations

We cannot rule out that infants might have experienced some stress during the sample collection however, any bias introduced by this however would be independent of prenatal Pb exposure which occurred 1–2 years earlier and therefore could not confound our results. Our results are consistent with a maladaptive effect of prenatal Pb exposure on the HPA axis maturation but the implications of this must still be assessed. Our study considered only one cortisol rhythm measure, and longitudinal measures of salivary cortisol rhythms beyond 2 years of age may shed further light on this developmental trajectory.

## Conclusions

To our knowledge, this is the first human study to look at the association of prenatal Pb exposure and infant basal diurnal cortisol rhythms as an index of HPA axis functioning. We found that age predicts lower basal cortisol secretion and that early prenatal Pb exposure is associated with dysregulated infant HPA axis function perhaps representing premature HPA axis maturation. More research is needed to confirm these findings. Long-term follow-up of these PROGRESS infants will allow additional insights into the possible health effects resulting from prenatal exposure to Pb and subsequent HPA axis disruption.

## Additional file

Additional file 1: Details for statistical analysis method and Additional file 1: Table S1 "Salivary cortisol geometric means and sample characteristics by age group". (DOCX 18 kb)

## Abbreviations

2 T: second trimester of pregnancy
3 T: third trimester of pregnancy
AUC: area under the curve
BMI: body mass index
BPb: blood lead
CAR: cortisol awakening response
CI-95 %: confidence interval
CRYSIS: crisis in family systems
HPA: hypothalamic pituitary adrenal
IMSS: Instituto Mexicano del Seguro Social
Pb: lead
SD: standard deviation
SE: standard error
