# Supplementary material for: Longitudinal associations of age and prenatal lead exposure on cortisol secretion of 12–24 month-old infants from Mexico City
Source: Environ Health. 2016 Feb 29;15:41. doi: 10.1186/s12940-016-0124-1 (PMC4772459; doi:10.1186/s12940-016-0124-1)
Supplement: Additional file 1: — Details for statistical analysis method and Additional file 1: Table S1 "Salivary cortisol geometric means and sample characteristics by age group". (DOCX 18 kb) [file 12940_2016_124_MOESM1_ESM.docx]

**Supplemental Material**

**Title: Longitudinal associations of age and prenatal lead exposure on cortisol secretion of 12-24 month-old infants from Mexico City.**

**Authors:** Marcela Tamayo y Ortiz, Martha María Téllez-Rojo, Rosalind J. Wright, Brent A. Coull, Robert O. Wright.

**Statistical Methods**

We used functional mixed models [[1](#_ENREF_1)] to characterize the associations between Pb exposure and cortisol throughout the course of sampling day, controlling for potential confounders. We describe our methods in further detail as follows. For each subject *i* there are n*_i_* cortisol samples *y_i_*=(*y_i_*_1_, *y_i_*_2_,…., *y_ini_*) taken at different times since awakening *t*_1_*, t*_2_*,…, t_ni_*. The cortisol level at time *t* is

$$y_{it}=f_{i}\left( t \right)+ Ɛ_{it}, (1)$$

where *f_i_*(*t*) is the cortisol profile for each subject and a residual error Ɛ_it_ with a mean of zero and a variance of σ^2^.

These constant-effects model took the following form:

$$f_{i}{(t}_{ij})=\beta_{0}{(t}_{ij})+\beta_{1}{Pb}_{i}+\beta_{c}C_{i}+b_{i}{(t}_{ij}). (2)$$

The results included a parametric coefficient ($\beta_{1})$for Pb and a smooth intercept representing the main effect of time since awakening. In this constant-effect model, a negative coefficient for Pb reflects a downward constant shift of the curve, and therefore a decrease in the total diurnal cortisol, per unit increase in Pb exposure. Conversely, a positive coefficient reflects an upward constant shift of the curve, and therefore an increase in the total diurnal cortisol, per unit increase in Pb exposure. In the version of the model treating Pb as a categorical (low, moderate, high) exposure, the Pb term was replaced by two indicator variables, one corresponding to Pb exposure being between 5 and 10 µg/dl and another indicating Pb level greater than 10 µg/dl. In the constant effect models, these effects were assumed to be constant across the course a day.

In our second model, we allowed the shape of the mean cortisol curve over the course of the day to change in response to Pb exposure; for example, it could plateau shortly after awakening or increase in slope later during the day. In this model we can evaluate whether Pb exposure has an effect at a particular time of day, which would be missed in the first constant effect model. For a general lead exposure term ${Pb}_{i},$the model takes the form

$$f_{i}{(t}_{ij})=\beta_{0}{(t}_{ij})+\beta_{1}{(t}_{ij}){Pb}_{i}+\beta_{c}C_{i}+b_{i}{(t}_{ij}). (3)$$

For these models, we present the estimates, and associated 95% pointwise confidence intervals, of the Pb effect $\beta_{1}{(t}_{ij})$graphically, since these estimated curves highlight the portions of the day in which Pb exposure is associated with cortisol levels. As for the time-constant models, we ran the time-varying effect models both for continuous and categorical (low, moderate, high) versions of the Pb exposures.

| **Table 1. Salivary Cortisol Geometric Means and Sample Characteristics by Age Group** | | |
| --- | --- | --- |
|  | **12 month infants** | **18-24 month infants** |
|  | *n=255* | *n=150* |
| **Cortisol concentration (nmol/L), Geometric mean** | | |
| Sample 1 | 12.4 | 11.0 |
| Sample 2 | 6.0 | 5.0 |
| Sample 3 | 4.9 | 4.4 |
| Sample 4 | 2.2 | 1.7 |
| **Time since wake up (hr:min), Mean (SD)** | | |
| Sample 1 | 00:33 (00:25) | 00:34 (00:29) |
| Sample 2 | 03:56 (01:01) | 03:59 (01:05) |
| Sample 3 | 08:03 (01:13) | 08:12 (01:18) |
| Sample 4 | 12:46 (01:07) | 12:55 (01:09) |
| **Sampling day on weekend (vs weekday)** | | |
| First sampling day n (%) | 62 (24) | 41 (28) |
| Second sampling day n (%) | 54 (21) | 26 (18) |
| Sample 1: after wake up and diaper change, before breakfast. Sample 2: between 11:00 am and1:00 pm). Sample 3: between 3:00-5:00 pm. Sample 4: at least 30 min after dinner, before bed time. | | |

References

1. Sanchez BN, Wu M, Raghunathan TE, Diez-Roux AV: **Modeling the salivary cortisol profile in population research: the multi-ethnic study of atherosclerosis**. *American journal of epidemiology* 2012, **176**(10):918-928.
